# Novel peptide probes to assess the tensional state of fibronectin fibers in cancer

**DOI:** 10.1038/s41467-017-01846-0

**Published:** 2017-11-27

**Authors:** Simon Arnoldini, Alessandra Moscaroli, Mamta Chabria, Manuel Hilbert, Samuel Hertig, Roger Schibli, Martin Béhé, Viola Vogel

**Affiliations:** 1 Laboratory of Applied Mechanobiology, Institute of Translational Medicine, Department of Health Sciences and Technology, ETH Zurich, Vladimir-Prelog-Weg 4, 8093 Zurich, Switzerland; 2 Center for Radiopharmaceutical Sciences, Paul Scherrer Institute, OIPA/103, 5232 Villigen PSI, Switzerland; 3 Laboratory of Biomolecular Research, Paul Scherrer Institute, OFLC/102, 5232 Villigen PSI, Switzerland; 4 Hertig Visualizations, Technikumstrasse 10B, 3400 Burgdorf, Switzerland; 5 Institute for Pharamaceutical Science, Department of Chemistry and Applied Biosciences, ETH Zurich, Vladimir-Prelog-Weg 4, 8093 Zurich, Switzerland

## Abstract

Transformations of extracellular matrix (ECM) accompany pathological tissue changes, yet how cell-ECM crosstalk drives these processes remains unknown as adequate tools to probe forces or mechanical strains in tissues are lacking. Here, we introduce a new nanoprobe to assess the mechanical strain of fibronectin (Fn) fibers in tissue, based on the bacterial Fn-binding peptide FnBPA5. FnBPA5 exhibits nM binding affinity to relaxed, but not stretched Fn fibers and is shown to exhibit strain-sensitive ECM binding in cell culture in a comparison with an established Fn-FRET probe. Staining of tumor tissue cryosections shows large regions of relaxed Fn fibers and injection of radiolabeled ^111^In-FnBPA5 in a prostate cancer mouse model reveals specific accumulation of ^111^In-FnBPA5 in tumor with prolonged retention compared to other organs. The herein presented approach enables to investigate how Fn fiber strain at the tissue level impacts cell signaling and pathological progression in different diseases.

## Abstract (web summary)

The extracellular matrix is under variable strain, but we lack the tools to detect differences in strain. Here the authors develop a probe based on a bacterial fibronectin-binding peptide that binds to relaxed fibronectin fibrils and detects relaxed matrix in cell culture, tissue slices and *in vivo*.

## Introduction

Despite major progress in our understanding how proteins act as mechano-chemical switches^1–4^ and how the mechanobiology of cells regulates their fate^5–8^, our knowledge about the mechanobiology of tissues in development, under homeostatic conditions and in pathological transformations remains sparse. Even though a large toolbox of technologies to quantify forces and mechanical strains at the molecular and cellular level is available today^8–10^, how to translate these findings to the tissue level is a challenge, since not a single probe to either measure forces or force-induced molecular strains in tissues exists today that is applicable to be used in vivo or in histological tissue sections. This prevents a thorough characterization of how the tensional states in tissue fibers might tune the structure–function relationships of extracellular matrix (ECM) proteins, and how this might ultimately regulate tissue growth, regeneration, homeostasis or disease progression. For cancer^11–13^ and fibrotic disorders^14,15^, altered cellular forces, changed ECM composition and crosslinking, and thus increased tissue stiffness are well documented, yet, how this correlates with the local strains of ECM fibers and thus the ECM-cell crosstalk remains unknown. Availability of novel probes to document and track ECM fiber strain in histological samples and living tissues is thus highly relevant. Fibronectin (Fn) represents one of the most abundant ECM proteins^16,17^ with a plethora of binding sites for other ECM proteins, growth factors and cells, and especially its isoforms containing the extra domains A (EDA) and B (EDB), are overexpressed in cancer, wound healing and inflammation^17–19^, and serve as biomarkers for metastasis and bad prognosis in many cancer types^20–24^. While major mechano-regulated alterations in Fn’s structure–function relationship have been reported^3,25–27^, how many of its binding sites, including the growth factor binding sites^28–31^, are regulated by mechanical tension acting on Fn fibers remains unknown. The strain of ECM fibers depends on both, cellular force application to individual Fn fibers as well as on the overall composition of the ECM^3–5,8,11,32–34^. Even though our focus here is on cancer, the availability of new mechanosensitive strain probes will be equally important to finally uncover how forces regulate tissue growth and regeneration processes, from early development to wound healing, where fibronectin expression and matrix assembly is highly upregulated as well^17,35^.

**Fig. 1:**
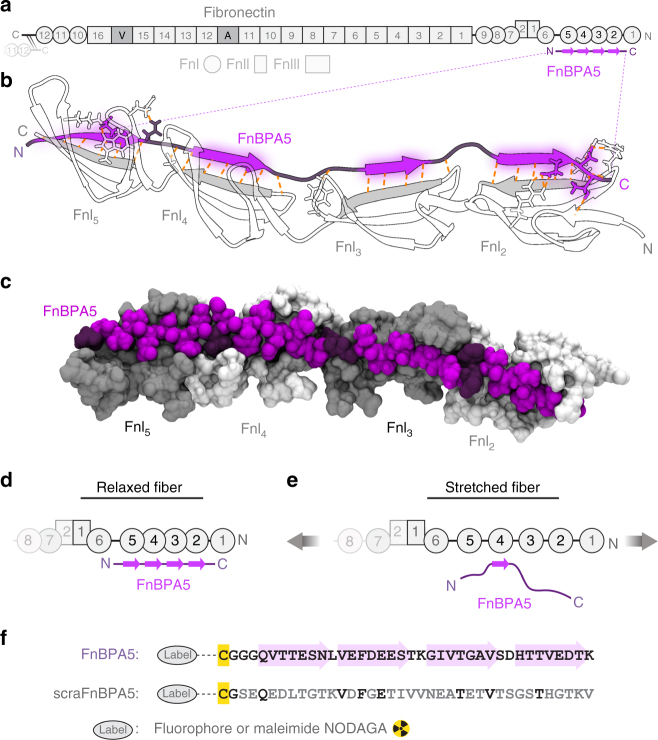
Multivalent binding motif by which the bacterial peptide FnBPA5 specifically recognizes the N-terminal fibronectin (Fn) type I domains (FnI). **a** Schematic representation of the multidomain protein Fn. The binding epitope for FnBPA5 (purple) is located near the N-terminus of fibronectin (gray). **b** FnBPA5 binds to modules FnI_2–5_ (white, strands forming intermolecular β-sheets in gray) via an antiparallel beta-zipper by forming an extensive network of backbone hydrogen bonds (orange dotted lines). Linker residues of FnBPA5 that connect the β-strands are shown in darker purple. **c** Surface rendering of the complex shown in **b**, highlighting the snug fit of the interaction. Odd-numbered FnI modules are shown in darker gray for distinction. **d** FnBPA5 binds to relaxed, but not to stretched Fn fibers, as fiber stretching causes a structural mismatch with its multivalent binding epitope located on the Fn type I domains FnI_2–5_. **e** Stretching of Fn leads to a reduction of multivalency and thus an affinity switch to low binding of FnBPA5. **f** Sequences of the FnBPA5 peptide and its randomly scrambled negative control, scraFnBPA5. Residues in the wild type peptide forming intermolecular β-sheets are indicated with light purple arrows, and residues of the scrambled control peptide conserved with respect to the wild type are shown in black. Both peptides were labeled on the N-terminal cysteine residues (yellow) with Alexa488 or Cy5 fluorophores for in vitro experiments and NODAGA for in vivo experiments

Here, we explore whether properly designed peptide probes can be exploited to identify the tensile state of ECM fibers in histological tissue sections, as well as in living animals. Even though we had introduced a molecular probe in 2001 that recognizes the mechanical strain of Fn fibrils based on fluorescence resonance energy transfer (FRET)^25,33,34^, quantifying Fn-FRET ratios is more complicated in tissues compared to cell culture experiments. Instead, we asked whether Fn-binding peptides (FnBPs) derived from bacterial adhesins can be exploited to distinguish highly stretched from relaxed Fn fibers in vivo, as well as to stain the fiber strain of Fn in histological samples. Bacteria evolved adhesins that specifically target fibronectin with high affinity^36^. These FnBPs are unstructured in solution and form an antiparallel tandem β-zipper upon binding in a row to multiple N-terminal fibronectin type I (FnI_1–5_) modules. The tight binding complex formed with FnI modules is stabilized by backbone hydrogen bonds and is connected via short unstructured peptide loops (Fig. 1)^27,37,38^. This multivalent binding to multiple adjacent domains greatly increases the binding affinity to Fn, from the μM to the nM range, if targeting not only FnI_4–5_ but FnI_2–5_, respectively^37–39^. We could show previously that the spatial match between this multivalent binding motif of the short peptide fragment STAFF5C targeting FnI_4–5_ derived from *Staphylococcus aureus* (FnBPA5) is destroyed by stretching Fn fibers^27,37^. Here, we validate the use of the much longer FnBPA5 peptide which specifically binds to relaxed but not stretched Fn fibrils, as schematically shown in Fig. 1d, e, to probe the strain of Fn fibers at the tissue level.

For the histological tissue stains, as well as for the in vivo experiments, a mouse model for human prostate carcinoma was chosen (PC-3 xenografts). This type of carcinoma exhibits an extended tumor microenvironment, caused by a dysregulation of the equilibrium between matrix production and turnover, representing an adequate model for the validation of the FnBPA5 peptide probe^40^. To provide a proof-of-concept, histological tissue sections of cancer stroma were co-stained with a polyclonal Fn antibody, as well as with the fluorescently labeled FnBPA5 peptide (Fig. 1f), to image the locations of total Fn vs. relaxed Fn, respectively. To then investigate its suitability as an in vivo probe, the pharmacokinetics of ^111^In-FnBPA5 and its distribution in various organs was explored in living mice after injection into the tail vein using single photon emission computed tomography (SPECT) analysis.

## Results

### Use of FnBPA5 to assess the mechanical tension of Fn fibers

To systematically explore whether peptides can be utilized as strain probes to visualize the mechanical tension of ECM fibers in histological tissue sections and in living animals, we tested the synthesized bacterial Fn binding peptide FnBPA5, together with a scrambled peptide (scraFnBPA5) as negative control having the same amino acid residues and could show that it has no affinity to Fn (Fig. 1d). For the in vitro analysis and physicochemical characterization, the peptides were functionalized with an Alexa- 488 or Cy5 fluorophore for immunofluorescence microscopy studies, while they were radiolabeled with ^111^In for the in vivo SPECT/CT imaging and the biodistribution analysis performed in a prostate cancer mouse model.

### FnBPA5 preferentially binds to relaxed Fn fibers

**Fig. 2:**
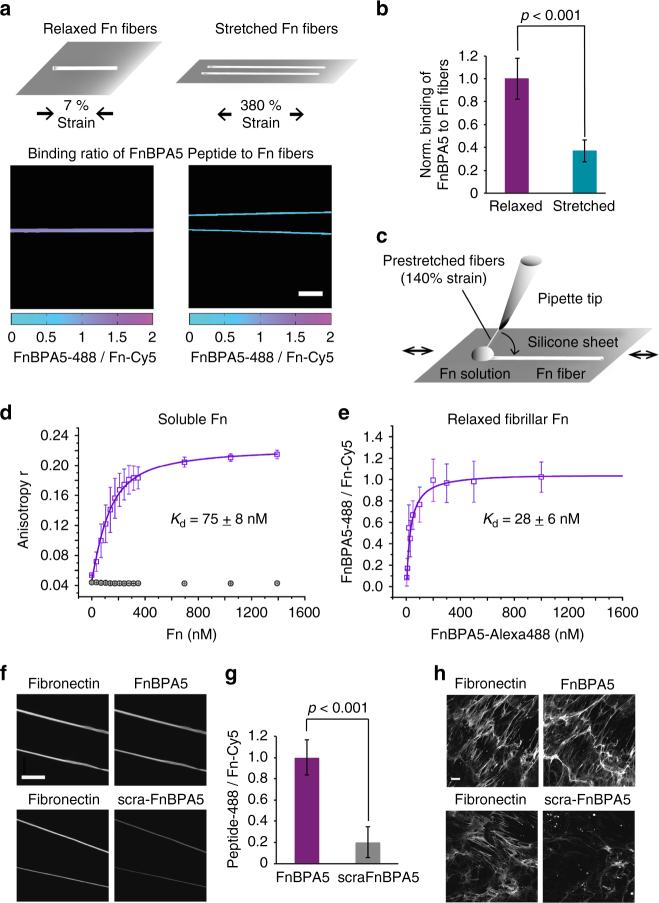
In vitro binding studies of the peptide FnBPA5-Alexa-488 and the scrambled control (scraFnBPA5-Alexa-488) to soluble and fibrillar Fn. **a** Mechanosensitive binding of FnBPA5 was tested using relaxed (7% strain) and stretched (380% strain) fibers. Color-coded intensity ratio images of FnBPA5-Alexa488 divided by Fn-Cy5 of manually pulled Fn fibers of different mechanical state show higher binding to relaxed fibers than to stretched ones, leading to a higher FnBPA5/Fn ratio (representative images). Scale bar, 50 μm. **b** Normalized analysis of relaxed and stretched fibers showing a significant decrease in binding ratio of FnBPA5 from relaxed to stretched fibers. Data from 30 fibers from three independent experiments with error bars being standard deviations. *p*-value was obtained from a Student’s *t* test. **c** Schematic of manual fiber pulling, using a sharp pipette tip dipping into a Fn solution and pulling Fn fibers onto a flexible silicone sheet. **d** The binding of FnBPA5-Alexa-488 and scraFnBPA5-Alexa488 to soluble plasma Fn was determined using an anisotropy measurement (Methods). The results show a *K*_d_ of 75 ± 8 nM for FnBPA5-Alexa488 and no specific binding for the scrambled control peptide. **e** Measurements of affinity of FnBPA5-Alexa488 to single relaxed Fn fibers showed a *K*_d_ of 28 ± 6 nM. **f** Fluorescence images showing the binding of FnBPA5-Alexa488 and scraFnBPA5-Alexa488 to single relaxed Fn fibers (7% strain) using a previously described stretch assay^41^. For this experiment Cy5-labeled Fn was used (images on the left side). Scale bar, 50 μm. **g** The quantification of the fluorescence intensity showed a significant higher binding of FnBPA5-Alexa488 compared to the control. Data from 30 fibers from three independent experiments were analyzed. Shown error bars represent the standard deviation. *p*-value was obtained from a Student’s *t* test. **h** Human dermal fibroblast ECM was stained for Fn and incubated with FnBPA5-Alexa488 and the scrambled control peptide, respectively. Representative images show specific binding of FnBPA5-Alexa488 to Fn. Scale bar, 10 μm

To confirm strain-dependent binding of the FnBPA5 peptide to fibrillar Fn in vitro, and since only its shorter fragment STAFF5C was tested earlier^37^, single Fn fibers were pulled out of a concentrated Fn solution containing 5% Cy5-labeled Fn and deposited onto a stretchable silicone sheet to adjust a defined mechanical Fn fiber strain (Fig. 2)^41^. Representative ratiometric images of fluorescent signals from the peptide vs. Fn, i.e., FnBPA5-Alexa 488 vs. Fn-Cy5, respectively, show significantly higher peptide binding of FnBPA5 to relaxed Fn fibers compared to stretched fibers (Fig. 2a). The quantification of three independent experiments showed a significant (*p* < 0.001, Student’s *t* test) decrease in FnBPA5/Fn ratio for stretched fibers (380% mechanical strain) compared to relaxed ones (7% mechanical strain) (Fig. 2b). This indicates that stretching of individual Fn fibers destroys the multivalent binding motif as stretching causes a structural mismatch between the peptide and its multivalent binding epitope located on the Fn type I domains FnI_2–5_. This is in line with previously published computational results for FnBPA5, as well as with experimental binding studies using much shorter fragments of FnBPA5, i.e., the bivalent fragment of FnBPA5, STAFF5C binding to FnI_4–5_, and other bacterial and engineered FnBPs^27,37^.

### FnBPA5 binds with nM affinity to soluble and relaxed Fn

Affinity of FnBPA5-Alexa 488 towards relaxed fibrillar and soluble Fn was determined in vitro by two independent assays: fluorescent anisotropy titrations and single fiber binding assays. Fluorescence anisotropy titrations with FnBPA5-Alexa 488 revealed a dissociation constant (*K*_d_) of 75 ± 8 nM (mean ± standard deviation (SD), *n* = 3) to soluble Fn. In contrast, the control peptide scraFnBPA5-Alexa488 did not show any binding (Fig. 2d). Single Fn-fiber assays^41^ were then used to quantify the affinity of FnBPA5-Alexa488 and of its scrambled control peptide to relaxed Fn fibers. The saturation binding curve was derived from a pixel-by-pixel analysis of the intensity ratio of FnBPA5-Alexa 488 vs. Fn-Cy5-labeled fibers. Fitting of the binding curve revealed a *K*_d_ value of 28 ± 6 nM (mean ± SD, *n* = 3) (Fig. 2e). Confocal microscopy images of Fn fibers exposed to FnBPA5-Alexa 488 or its scrambled control peptide are shown in Fig. 2f. FnBPA5-Alexa-488 showed significantly higher binding to Fn fibers than the scrambled control peptide, confirming the sequence-specificity of the interaction (Fig. 2g). A displacement assay using ^nat^In-FnBPA5 (cold labeled) against FnBPA5-Alexa- 488 was performed to assess whether radiolabeling impairs FnBPA5 binding to single Fn fibers, showing that the radiolabeling procedure did not affect binding properties of FnBPA5 to Fn (Supplementary Fig. 1).

The dissociation constants for soluble, full-length Fn, and fibrillar Fn are close to the affinity reported for FnBPA5 binding to N-terminal Fn fragments in solution (*K*_d_ = 44.2 nM)^39^. Most importantly, the affinities of the FnBPA5-Alexa 488 to plasma Fn and to relaxed fibrillar Fn are comparable to, and of the same order of magnitude, as those reported for several clinical antibodies that target ECM proteins^42^. To test if similar tight binding is observed in native ECM, Fn-rich ECM assembled by human dermal fibroblasts was incubated for 1 h with FnBPA5-Alexa488 or scrambled control peptide. Specific binding to fibrillar Fn was only observed for FnBPA5-Alexa488 (Fig. 2h). To see whether FnBPA5 binding to Fn in cell culture also exhibits strain-sensitive behavior, we next compared it with our strain-sensitive Fn-FRET sensor^25,34^.

### Validation of FnBPA5 in ECM by comparison to a Fn-FRET probe

**Fig. 3:**
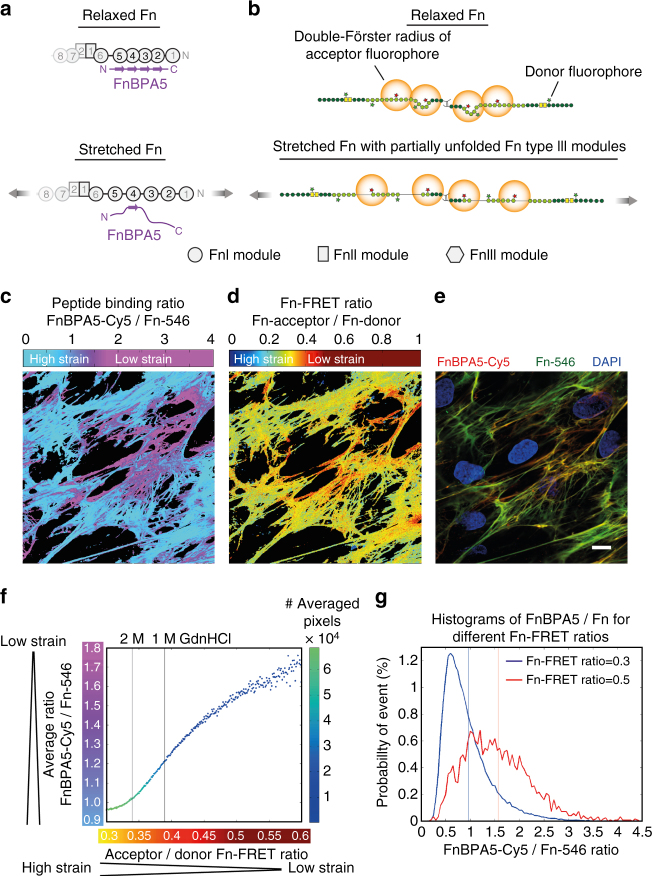
Comparison of FnBPA5 binding to a fibroblast-grown native Fn matrix using a Fn-FRET probe. **a** Schematic of strain-sensitive binding of FnBPA5 to N-terminal FnI domains of Fn. **b** Schematic of Fn-FRET probe with acceptor fluorophores at specified domains and donor fluorophores at random locations, exhibiting a lower energy transfer in stretched ECM fibers compared to relaxed ones. **c** FnBPA5-Cy5 was normalized with directly excited Fn-546, showing in the false color ratiometric image high FnBPA5 binding in violet areas. **d** False color ratiometric image of Fn-Acceptor 546/Fn-Donor 488 (FRET ratio). Red colors represent higher FRET ratios representing more relaxed Fn conformations, whereas blue colors show lower FRET ratios corresponding to regions with more stretched Fn. **e** Merged fluorescence image of FnBPA5-Cy5 (red), Fn-546 (green), and DAPI (blue) from the same field of view as in **c** and **d**. Scale bar, 10 μm. **f** Quantitative analysis of average FnBPA5 binding vs. Fn-FRET ratios for all images (*n* > 10) of an experiment shows an increase in FnBPA5 binding for higher FRET ratios. Number of pixels used for averaging is given in the color code. **g** Histograms of the FnBPA5-Cy5/Fn-546 ratios for two selected Fn-FRET ratios (0.3 and 0.5). Vertical lines represent the arithmetic means of the distributions. Shift of distribution of FnBPA5/Fn for the FRET value of 0.5 (red) indicates a higher overall binding compared to distribution for FRET value of 0.3 (blue). Please note that the FnBPA5-Cy5/Fn-546 ratios are intensity ratios and do not give the ratios of molecules

We next asked how well the FnBPA5 binding to Fn fibrils (Fig. 3a) within the ECM assembled by fibroblasts in cell culture correlates to the Fn fiber strain as probed by a totally different method, i.e., by using our well established Fn-FRET probe (Fig. 3b), which we broadly exploited before to visualize how cells stretch Fn fibers in 2D cell culture, cell-derived ECM scaffolds and in maturing microtissues^25,33,34,43,44^. Human dermal fibroblasts were cultured for 48 h and allowed to assemble their own ECM in the presence of Fn-FRET probes supplemented to the medium. One hour prior to fixation FnBPA5-Cy5 was added to the medium. Pixel-by-pixel analysis of normalized FnBPA5 binding to Fn fibrils (FnBPA5-Cy5 channel vs. directly excited acceptor channel of Fn-546) (Fig. 3c) vs. the Fn-FRET ratios (Fig. 3d) shows a significant spatial correlation between locations high in FRET ratios and high in peptide binding (Fig. 3c–e). This confirms that the FnBPA5 peptide preferentially binds to relaxed Fn fibers in cell culture. This is of particular interest, since the FnBPA5 peptide binds to and probes the N-terminal region of Fn, while the acceptors are localized at cryptic cysteines on the Fn type III modules, FnIII_7_ and FnIII_15_, respectively (Fig. 3a, b). To quantitatively test this correlation, the average FnBPA5/Fn ratios for all pixels sharing the same Fn-FRET ratio for multiple fields of view (*n* > 10) was plotted in Fig. 3f, with a color-code for the number of pixels used for the averaging. This analysis shows an increase of the average FnBPA5-Cy5/Fn-546 ratio with increasing Fn-FRET ratios, confirming the first impression of the image comparison by eye. In Fig. 3g, probability density functions of FnBPA5/Fn ratios for two different FRET ratios (0.3 in blue, 0.5 in red) are shown with vertical lines representing the arithmetic mean of the individual distributions. The correlation of the means using both probes, together with the pronounced shift of the two density functions with respect to each other validates that FnBPA5 binds predominantly to relaxed Fn fibers. In contrast, the scraFnBPA5 peptide did not bind to the ECM of human dermal fibroblasts made in cell culture (Fig. 2h). FnBPA5 can thus be exploited as a new tool to probe the mechanical strain of Fn fibers in settings that are not amenable to currently available methods, such as within histological tissue sections or in vivo.

### Tumor stroma contains large regions of relaxed Fn fibers

**Fig. 4:**
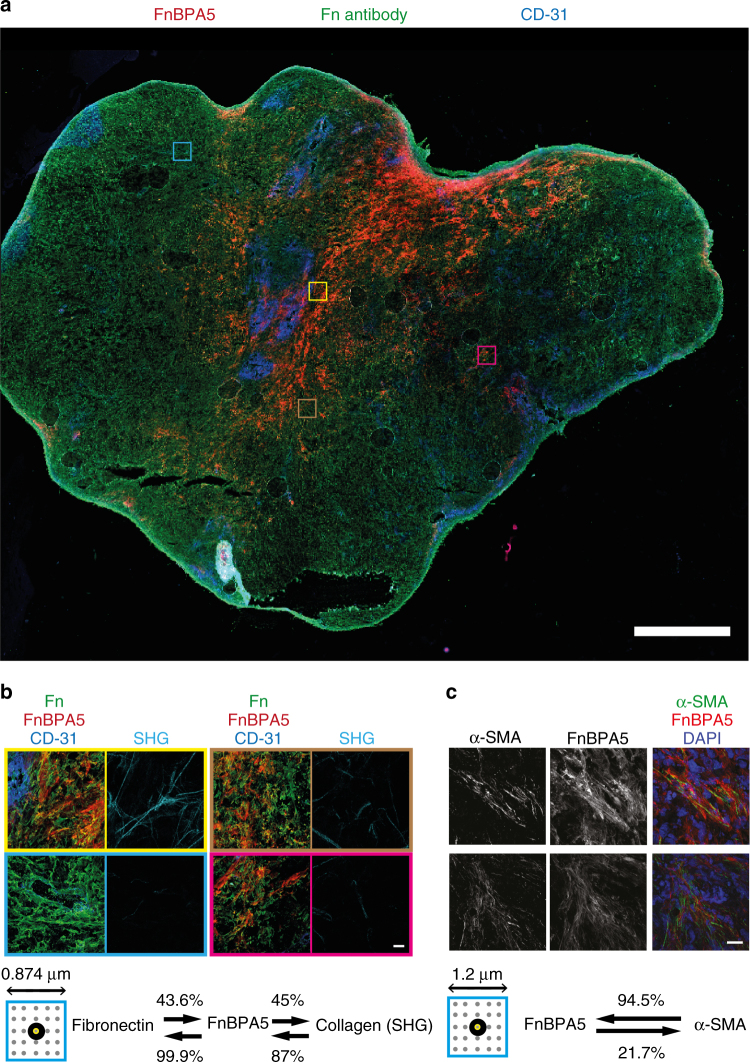
Post mortem tumor cryosection from prostate cancer (PC-3) xenografts. **a** Overview mosaic image of a whole PC-3 tumor tissue section co-stained with the peptide FnBPA5 Alexa-488 (red), together with polyclonal antibodies against Fn (green) and endothelial cell marker CD-31 (PECAM-1) (blue). Scale bar, 1 mm **b** Zoomed-in high-resolution z-projection images of different indicated regions showing superpositions of the stains FnBPA5 (red), fibronectin (green), and CD-31 (blue), as well as the mature collagen fibers in the same regions as visualized by SHG (cyan). Analysis of neighboring pixels (indicated by the small box showing the 5 × 5 pixel matrix surrounding each analyzed pixel) between antibody-stained Fn, FnBPA5 peptide and collagen bundles (SHG) showed a clear spatial proximity of FnBPA5 with Fn (99.9% of all FnBPA5 pixels have at least one neighboring Fn pixel). A total of 87% of pixels from mature collagen bundles were found to be in close proximity (within the 5 × 5 matrix of neighboring pixels) of FnBPA5-positive pixels (19 images analyzed). Scale bar, 20 μm. **c** Representative z-projection images of PC-3 tumor tissue sections stained for α-SMA and FnBPA5 and merged with nuclei stainings using DAPI. Analysis of neighboring pixels (indicated by the small box showing the 5 × 5 pixel matrix surrounding each analyzed pixel) reveals that 94% of the α-SMA-positive pixels are in close proximity (within the 5 × 5 matrix of neighboring pixels) to FnBPA5-positive regions (60 images analyzed). Lower thresholds were set based on individual channels to exclude unspecific pixels from the analysis, and a 5 × 5 matrix was used for the spatial proximity analysis of each pixel in 60 images, respectively, with each of the analyzed pixels being in the center (Methods section for more details). Scale bar, 20 μm

To gain insights into the mechanical strain of Fn fibers in tumor tissue, post mortem tumor cryosections from human prostate cancer (PC-3) xenografts were co-stained with FnBPA5-Alexa488 and with various antibodies. Staining with a polyclonal Fn antibody to visualize the overall Fn distribution over the whole tissue section (Fig. 4a) revealed that Fn is abundantly present, as expected^45^. In contrast, the FnBPA5-Alexa488 stain is highly heterogeneous whereby only some regions but not others show that Fn exists in more relaxed conformations. A spatial proximity analysis using 19 images of maximum intensity z-projections, revealed that 99.9% of the FnBPA5-positive pixels were in close spatial proximity with antibody-stained Fn pixels, while only 43.6% of Fn pixels had FnBPA5 pixels within their neighboring 5 × 5 evaluation matrix (Fig. 4b). To further confirm specific binding of FnBPA5 to Fn fibers in the cryosections, several grayscale intensity line profiles are given for two representative high-resolution single slice confocal images taken at the FnBPA5 and Fn antibody channels, respectively, as well as the pixel-by-pixel intensity rations in false colors as shown in Supplementary Fig. 2.

Since this is a surprising observation and since nothing is known so far about potential mechanisms that regulate the tensile state of Fn fibers in tissues, we next tried to establish first functional correlations. To first address whether the relaxed Fn conformations are mostly found around the tumor vasculature, the endothelial cells were stained using a CD-31 (PECAM-1) antibody. In agreement with the literature, regions of high and poor vascularization are seen^46,47^. Significantly though, highly vascularized regions do not typically co-localize in these sections with the regions rich in FnBPA5 peptide as seen in the overview image (Fig. 4a) and in the high-resolution zoomed-in images (Fig. 4b).

Second, we had previously demonstrated that relaxed, but not stretched, Fn fibers serve as initial template to nucleate the onset of collagen I fiber assembly in cell culture and that Fn fibrils are partially relaxed in the presence of a collagen I rich ECM and revert to stretched fibers if collagen was enzymatically degraded^4^. We therefore analyzed here whether the FnBPA5 peptide is preferentially found in regions rich in mature collagen fibrils, as quantified by second harmonic generation (SHG). The SHG signals from the same regions show indeed that FnBPA5 peptide-rich regions are also rich in collagen bundles. A spatial proximity analysis revealed that 87% of all collagen bundle pixels as seen by SHG are in close spatial proximity to one or more pixels of FnBPA5 (as defined by the schematic squares in Fig. 4b and c and explained in the Materials and Methods section), whereas only 45% of the FnBPA5-positive pixels are in proximity to SHG-positive collagen pixels (Fig. 4b).

Third, since tumor stroma is enriched by more contractile myofibroblastic phenotypes^48^, the cryosections were co-stained with FnBPA5-Cy5 and the myofibroblastic marker anti-alpha smooth muscle actin (α-SMA) (Fig. 4c). Perhaps surprisingly, we could observe a higher binding of FnBPA5 peptides in the ECM surrounding α-SMA-expressing cells (Fig. 4c). The differentiation of fibroblasts towards the more contractile myofibroblasts is induced by upregulated levels of the transforming growth factor-β (TGF-β)^48,49^. Since myofibroblasts also show an upregulated expression of Fn and collagen I and subsequent ECM assembly in cell culture and at the tissue level^48^, we asked whether the correlation between upregulated α-SMA and FnBPA5 peptide binding can be recapitulated in cell culture. Indeed, the ECM of human dermal fibroblasts cultured for 5 days in medium supplemented with 40 pM TGF-β showed significantly higher binding of FnBPA5 (Supplementary Fig. 3a), as well as increased Fn expression levels, as observed by Western blotting compared to the untreated control (Supplementary Fig. 3b) in agreement with the literature^50^. Densitometric quantification of blots from three independent experiments showed indeed a significant 2.3-fold increase in Fn normalized to the loading control GAPDH (Supplementary Fig. 3c), similar to the increase of FnBPA5 binding observed via fluorescence measurements by a plate reader. The blot furthermore showed upregulated α-SMA expression for TGF-β treated fibroblasts (Supplementary Fig. 3b). Taken together, this suggests that the presence of myofibroblasts might cause an upregulated collagen I assembly into thick collagen fiber bundles, as detected by SHG, which might then lead to a relaxation of Fn fibers as previously seen in cell culture^4^. Additionally or alternatively, some plasma Fn might adsorb to the thick collagen bundles as previously seen in fibroblast contracted collagen gels^44^.

### High plasma stability makes FnBPA5 suitable for in vivo use

The stability of peptides in blood is often limited as they are rapidly cleaved by catalytic enzymes found in tissue and blood, resulting in a short biological half-life. This can significantly limit their in vivo application^51^. To verify whether the FnBPA5 peptide can be used for in vivo applications, we next performed a plasma stability test. The metabolic stability of the peptide radiotracer ^111^In-FnBPA5 was tested in vitro by incubating the radiolabeled FnBPA5 at 37°C with human blood plasma, or water as control. The unbound peptide fraction was separated from the plasma proteins at different time points (0, 1, 24, 48, and 72 h) and was analyzed by reversed-phase high-pressure liquid chromatography (RP-HPLC). Approximately 80% of ^111^In-FnBPA5 was still intact after 72 h (Supplementary Fig. 4), thus verifying that the radiotracer ^111^In-FnBPA5 has sufficient plasma stability to be used for in vivo applications.

### ^111^In-FnBPA5 accumulates in prostate tumor xenografts

**Fig. 5:**
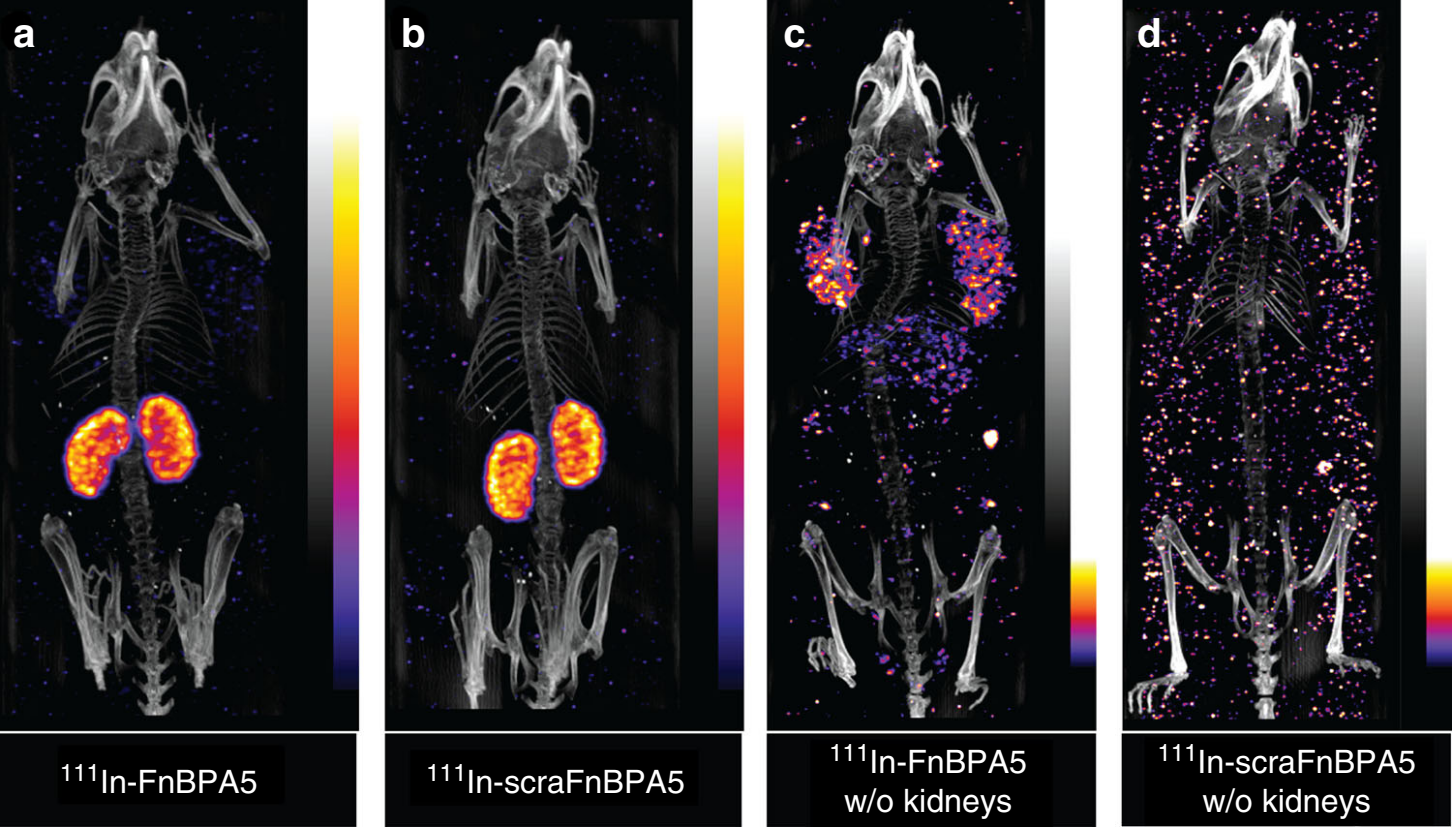
SPECT/CT images of mice bearing PC-3 xenografts 96 h post injection. Mice were injected with 15 MBq ^111^In-FnBPA5 **a**, **c** ^111^In-scraFnBPA5 **b**, **d**, respectively. Images were acquired post mortem 96 h post injection. In **a**, **b** dominant kidney uptake of both ^111^In-FnBPA5 and scrambled control peptide indicate unspecific clearance via the kidney. To assess additional information on peptide distribution within the body, kidneys were removed **c** and **d**. In **c** uptake in tumors and liver for ^111^In-FnBPA5 is shown, whereas the ^111^In-scraFnBPA5 control does not show any specific uptake in other organs. Results of SPECT/CT scans from individual subfigures are also shown in Supplementary Movies 1–4 showing 3D rotation movies of the respective scans

In vivo SPECT/CT analysis was conducted to monitor the distribution of ^111^In-FnBPA5 up to 96 h post injection (p.i.). PC-3-bearing mice, a subcutaneous model for human prostate carcinoma, were injected with approximately 15 MBq ^111^In-FnBPA5 and ^111^In-scraFnBPA5 (2.4 nmol, 100 µL PBS), respectively. ^111^In-FnBPA5 (Fig. 5a and Supplementary Movie 1) and the scrambled control peptide (Fig. 5b and Supplementary Movie 2) mainly accumulated in the kidneys. High renal uptake is a common observation in targeted radionuclide therapy with small peptides (e.g., somatostatin analogs)^52–54^. Using the scrambled control peptide, we excluded a Fn-specific binding in the kidneys. The high renal uptake could be related to megalin-mediated endocytosis in the proximal tubules, which is a physiological mechanism that ensures the re-uptake of valuable nutrients^53^.

To visualize additional binding of the radiotracers throughout the body, the kidneys were subsequently removed from the sacrificed mice and the scan was repeated. Mice injected with ^111^In-FnBPA5 showed activity in different organs, with a predominant uptake in the tumor, liver, and spleen (Fig. 5c and Supplementary Movie 3). In contrast, for mice injected with ^111^In-scraFnBPA5 no uptake was visible after kidney dissection (Fig. 5d and Supplementary Movie 4).

**Fig. 6:**
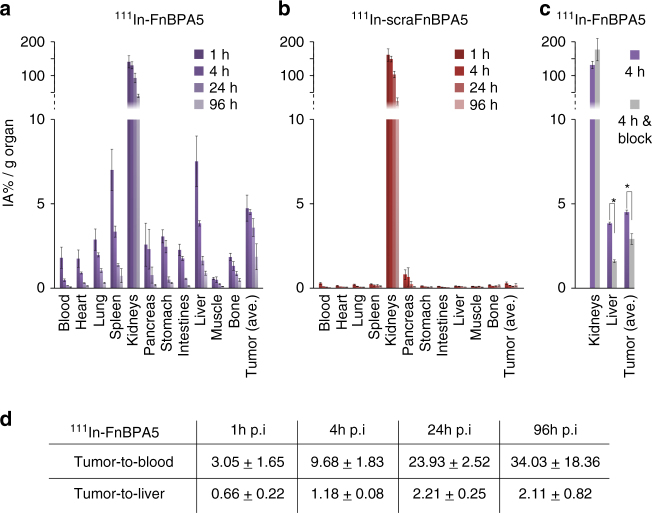
Biodistribution and blocking study of radiolabeled ^111^In-FnBPA5 and ^111^In-scraFnBPA5. **a** The biodistribution of ^111^In-FnBPA5 in PC-3-bearing mice was monitored at different time points in various organs. **b** The biodistribution of ^111^In-scraFnBPA5 was monitored in the same way. ^111^In-FnBPA5 (violet) showed a significant higher uptake than ^111^In-scraFnBPA5 (red) in all organs except for the kidneys, confirming the Fn-specificity of the accumulation. **c** Blocking studies were performed and the uptake was analyzed 4 h post injection (p.i.). The blocking of the binding sites via the pre-injection of unlabeled FnBPA5 caused a significant reduction in the uptake of ^111^In-FnBPA5 in both liver and tumor (*p < 0.05, Student’s *t* test, *n* = 4), whereas it did not change the uptake in the kidneys. The blocking showed a higher influence on ^111^In-FnBPA5 uptake in the liver than in the tumor, suggesting the presence of more binding sites (fibronectin) in the tumor. **d** The higher retention time of ^111^In-FnBPA5 in the tumor is reflected in increasing tumor-to-blood and tumor-to-liver ratios with increasing time. Bars represent means with error bars being standard deviations

To gain insights into tissue-specific peptide pharmacokinetics, groups of four PC-3-bearing mice were injected with ~150 kBq ^111^In-FnBPA5 and ^111^In-scraFnBPA5 (2.4 nmol per 100 µL PBS), respectively. The biodistributions of both tracers were analyzed at various time points (1, 4, 24, and 96 h p.i.) by means of percentage of injected activity per gram tissue (% IA per g). An equal accumulation of both peptides was observed in the kidneys (Fig. 6a, b), confirming the findings obtained from SPECT imaging. In both cases, maximal activity was seen at 1 h p.i. (140 ± 18% IA per g for ^111^In-FnBPA5 and 163 ± 18% IA per g for ^111^In-scraFnBPA5 (mean ± SD), *n* = 4 for each). In contrast to the scrambled control peptide, ^111^In-FnBPA5 showed additional accumulation in all other examined organs (Fig. 6a), particularly in the tumor, liver, and spleen. The values were significantly higher compared to the scrambled control peptide at all time points: the tumor uptake was significantly higher with a maximum at 1 h p.i. (4.74 ± 0.77% IA per g). Interestingly, the tumor-to-blood ratio increased from 3.05 ± 1.66 at 1 h p.i. to 34.03 ± 18.36 at 96 h p.i. and the tumor-to-liver ratio increased from 0.66 ± 0.22 at 1 h p.i. to 2.11 ± 0.82 at 96 h p.i. (Fig. 6d and Supplementary Table 1). This indicates that the retention of ^111^In-FnBPA5 in the tumor was significantly longer compared to other tissues (Fig. 6a).

To further confirm tumor stroma specific ^111^In-FnBPA5 uptake in vivo blocking experiments were performed. A 10-fold excess of unlabeled FnBPA5 (24 nmol) was pre-injected directly before ^111^In-FnBPA5 to saturate binding sites (Fig. 6c and Supplementary Tables 3 and 4). The pre-injection of unlabeled FnBPA5 caused a significant reduction (*p* < 0.05, Student’s *t* test) of ^111^In-FnBPA5 accumulation in all examined organs with exception of the kidneys and the pancreas (Supplementary Table 3). Furthermore, the blocking effect was less pronounced for the tumor tissue (−35.6%) than for the liver (−58.2%). In contrast, no significant differences were seen for ^111^In-scraFnBPA5 (Supplementary Table 4).

## Discussion

Major transformations of ECM accompany tumor progression^11,55^, yet how the reciprocal crosstalk between ECM and cells drives these processes remains unknown, at least in part due to the lack of probes to either measure cell traction forces in living tissues or the mechanical strain of ECM fibers. Fn is a key player within the ECM^17^ with Fn fiber assembly and functional display of binding sites for several binding partners changed by mechanical forces^3,27,56^. We introduce here the bacterial derived peptide FnBPA5^37^ and are able for the first time to distinguish different strains of Fn fibers in histological tissue sections (Fig. 4) and in a living mouse model (Fig. 5). In particular, we exploited FnBPA5 as nanoprobe to visualize regions rich in mechanically relaxed Fn conformations in cancer stroma (Figs. 4 and 6).

FnBPA5-Alexa488 shows strong binding affinity with a *K*_d_ in the low nanomolar range to plasma Fn (Fig. 2d) which is in agreement with in vitro binding studies of native FnBPA5 to N-terminal Fn fragments in solution^39^. The peptide also displays a *K*_d_ in the low nanomolar range for relaxed Fn fibers, which makes its specificity competitive to common monoclonal antibodies. The enhanced binding to relaxed, but not to stretched Fn fibers (Fig. 2a, b), confirms the high-resolution structural predictions from steered molecular dynamic simulations that showed that the multivalent epitope is destroyed upon fiber stretching^27,37^. The excellent correlation in cell culture between our two Fn fiber strain sensors, i.e., the FnBPA5 peptide and the Fn-FRET probe^25,34^ that both respond in different ways to stretch-induced structural alterations in the N-terminal FnI domains or the central FnIII domains, respectively, validates the strain-selective binding of the FnBPA5 peptide binding to native ECM fibers (Fig. 3).

Even though the importance of the physical properties of the microenvironmental niche is well recognized^1,7,9,12^, visualization of the tensional states of ECM fibers remains a dream. Mapping the ECM fiber strain for the first time in tumor tissue with the strain-sensitive FnBPA5 peptide as compared to using a polyclonal Fn antibody showed that Fn is abundant in this PC-3 tumor stroma, as expected^45^, but that FnBPA5 binds only to a fraction of all Fn fibrils (Fig. 4).

Tumor stroma is inherently heterogenous in its cellular composition, vascularization and ECM architecture and composition^13,47,48,55^. To ask whether the differences in ECM fiber tension and architecture correlate with differences in cell phenotypes, i.e., fibroblasts vs. myofibroblasts, we co-stained for the myofibroblast marker α-SMA and FnBPA5 (Fig. 4c) and found a substantial correlation. Analysis of multiple images revealed that 94.5% of the α-SMA-positive pixels where in close proximity to FnBPA5-positive pixels. This reciprocal relationship of FnBPA5 and α-SMA-positive pixels could be recapitulated in cell culture when stimulating the fibroblast to myofibroblast transition^48–50,57^ by the supplementation of TGF-β (Supplementary Fig. 3). Since myofibroblasts are known to drive the vicious cycle of collagen bundling and contraction^48,49^, we asked whether the ECM architecture is different between the regions that stain positively for FnBPA5 and those that do not. Indeed, 87% of all SHG-positive collagen bundle pixels were in proximity to FnBPA5 (Fig. 4b). Inversely, only 45% of the FnBPA5-positive pixels were in proximity to SHG-positive pixels which might be due to the existence of finer collagen fibrils not detected by SHG. Taken together, tumor stroma regions rich in myofibroblasts contain significant amounts of Fn in relaxed conformations. Since the conformational state of Fn regulates the exposure of its binding sites^3,27^, future work has to reveal how the tensional state of Fn fibers affects or even drives these pathological transitions.

Peptides to be used for in vivo applications require sufficient plasma stability, which often prohibited their use previously^58,59^. In contrast, our findings show that ^111^In-FnBPA5 is not rapidly degraded by enzymes in human blood plasma (Supplementary Fig. 4), as 80% of ^111^In-FnBPA5 was still intact after 72 h exposure to plasma in vitro. ^111^In-FnBPA5 thus has adequate serum stability for in vivo usage and a superior plasma stability compared to many other radiotracers^58,59^. Upon intravenous injection, SPECT/CT imaging revealed a Fn-specific uptake of ^111^In-FnBPA5 in PC-3 tumor stroma with an enhanced retention time in tumor tissue compared to other analyzed organs (Figs. 5 and 6). Considerable accumulation of peptide in the kidneys was observed (Figs. 5 and 6), as also typically seen for other peptide radiotracers^52,53^. Kidney uptake was not Fn-specific, as the accumulation was also seen for the scrambled peptide. For possible future in vivo applications, different strategies could reduce the renal uptake and thus possible related nephrotoxicity^60^. Uptake in liver, spleen, and tumor of ^111^In-FnBPA5 as revealed by in vivo SPECT images and post mortem biodistribution experiments was attributed to the specific binding properties of the FnBPA5 peptide to Fn, in contrast to the scrambled control. Indeed, liver and spleen uptake could be attributed to the intrinsic function of these organs as plasma Fn-excreting organ^61^ and as organ for blood filtering from pathogens^62^, respectively, with the peptide potentially being cargoed while bound to plasma Fn, as previously reported for other FnBPs^63^. The relatively fast clearance, evidence of liver and spleen uptake of plasma Fn from previous reports^64^ as well as the in vitro binding studies presented herein (Fig. 2d) further support this notion.

After 24 h post injection, 3.6% of the initially injected activity of ^111^In-FnBPA5 was detected per gram of tumor (Supplementary Table 1), which is comparable to in vivo uptake values of Fn-targeting antibodies^65^. The clearance of ^111^In-FnBPA5 from the tumor was considerably delayed compared to the other organs (Fig. 6). In particular, the tumor-to-background ratio progressively shifted towards higher values at later time points (Fig. 6 and Supplementary Table 1). This prolonged retention of ^111^In-FnBPA5 in cancerous tissue can be explained by a higher proportion of relaxed Fn conformations, an elevated level of total Fn or a higher amount of uptake of plasma Fn from the bloodstream, and thus an overall increase of available binding sites in the tumor microenvironment. Indeed, additional blocking experiments performed in vivo (Fig. 6c, Supplementary Tables 3 and 4) support this hypothesis as the blocking effect was less pronounced in the tumor compared to the liver. In addition to their high affinity and good plasma stability as illustrated here (Supplementary Fig. 4), peptides are expected to penetrate significantly better into dense stromal tissue structures compared to nanoparticles and antibodies because of their much smaller size^66^. While we had previously identified one antibody that binds preferentially to stretched Fn fibrils in a stretch assay^67^, peptides typically show much lower immunogenicity compared to antibodies, and solid-phase technology permits to chemically synthesize different ligands with a remarkable low production time and costs.

Taken together, our data provide a proof-of-concept illustrating how properly engineered peptides can be exploited to probe the mechanical strain of tissue fibers in histological tissue sections (Fig. 4), and that they accumulate in cancer tissue in living animals (Figs. 5 and 6). This provides the community with the urgently required tool needed to explore at the organ level how the reciprocal crosstalk between cells and their ECM is regulated by mechanical tension of ECM fibers, and how this might regulate homeostasis vs. disease progression. Also, the biomedical implications are highly significant, since nothing is known at the tissue level regarding the tensional state of Fn in healthy and diseased organs, nor during early development and aging. The availability of probes that can spatially map the strain of ECM fibrils, which is expected to be as heterogeneous as the cellular composition and organization, is crucial to learn how the mechanical strain of ECM fibrils correlates with alterations in localized cell signaling events, cell differentiation processes and disease progression^12,20^. Such knowledge is required to finally decipher how the crosstalk between cells and their ECM is regulated by fiber tension and how the concepts developed regarding ECM proteins acting as mechano-chemical switches^6,68^ co-regulate tissue growth, homeostasis, and pathologies.

These newly introduced peptides can easily be adopted to a range of specific biomedical needs by linking them to other labels, custom-built and adapted to case-specific requirements. In our case, the addition of a glycine spacer with a N-terminal cysteine rendered the bacterial Fn-targeting peptides FnBPA5 tunable to be linked to an additional signaling molecule such as an Alexa fluorophore or a chelator for radiolabeling (e.g. NODAGA), without blocking its binding properties. We thus anticipate that these and other novel strain-sensitive peptides, perhaps even targeting other ECM proteins, can be linked to radioisotopes or to other contrast agents allowing to image their distributions by different imaging modalities. Equally well, novel therapeutic approaches are feasible where relaxed Fn fibrils within ECM are specifically targeted, for example by linking such peptides to drugs or drug carrier systems to be used as therapeutic agents. Finally, these strain-sensitive peptides provide new tools for the design of in vivo pathology tests to assess whether and to what extent the tensional state of Fn within a tissue is altered. Using high affinity peptides to specifically target and visualize relaxed Fn in tissues, thus also opens the door for understanding the in vivo pathology of cancer and potentially many other diseases.

## Methods

### Fn isolation and labeling

Fn was isolated from human plasma (Zürcher Blutspendedienst SRK, Switzerland) using gelatin sepharose chromatography^69^. Plasma was thawed and passed through a PD-10 column (GE Healthcare, Germany) to remove aggregates. Effluent was collected and run through a gelatin sepharose column. After washing the column Fn was eluted from the gelatin column with a 6 M urea solution. Fn was then re-buffered to PBS before usage. For the labeling, Fn was denatured in a 4 M guanidinium hydrochloride (GdnHCl) solution to open up cryptic cysteines at FnIII_7_ and FnIII_15_ as described before^25^. Briefly, the denatured Fn was incubated with a 30-fold molar excess of Alexa Fluor 546 C5 maleimide (Invitrogen), for 1 h at room temperature. Labeled protein was then separated from free dye by passing it through a size-exclusion PD-10 column (GE Healthcare, Germany) into a 0.1 M NaHCO_3_ solution in PBS. For the second labeling step the protein was incubated for 1 h with a 70-fold molar excess of Alexa Fluor 488 carboxylic acid succinimidyl ester (Invitrogen) to label-free amines. Protein was then separated from dye using a PD-10 column and analyzed for concentration and donor to acceptor labeling ratio by measuring absorption at 280 and 488 and 546 nm. Used double-labeled Fn batch had an average of 7.5 donors and 3 acceptors per molecule. Single-labeled Fn was labeled as described above, but only involving the first labeling step using Cy5-maleimide (GE Healthcare).

### Chemical denaturation curve of FRET-Fn

For measurement of chemical denaturation curve of double-labeled Fn different denaturation mixtures with increasing concentration of denaturant GdnHCl was used to break open Fn’s secondary structure in solution^33^. To measure the denaturation curve an 8-well LabTek slide was covered with 2% bovine serum albumin (BSA) for 1 h rinsed with water, air-dried, and denaturation mixtures were added to the individual wells. The corresponding denaturation curve were measured using an Olympus FV 1000 confocal microscope with a PMT voltage of 550 V for both PMTs and can be found in Supplementary Fig. 5a.

### Synthesis and labeling of FnBPA5 and scrambled FnBPA5

The modified FnBPA5 and scrambled FnBPA5, shown in Fig. 1d, were commercially synthesized and HPLC-purified (Peptide Specialty Laboratories GmbH, Heidelberg, Germany, or Pichem GmbH, Graz, Austria). A spacer of three glycines and a cysteine residue at the N-terminus of the original peptide sequence from *S. aureus* were introduced. The cysteine was introduced for further modifications with fluorescent dye (Alexa-488 or Cy5) or with 1-(1,3-caroxypropyl)-1,4,7-triazacyclononane-4,7-diacetic acid (NODAGA), for radiolabeling with ^111^In. Lyophilized peptides were dissolved in water (TraceSELECT® quality, Sigma-Aldrich, Buchs, Switzerland) with 10% DMF and stored at −20°C for further usage.

### Fn fiber stretch assay

Fn fibers were manually pulled from a concentrated droplet of Fn in PBS (95% unlabeled and 5% Cy5-labeled protein) using a sharp pipette tip and deposited onto a flexible silicone sheet (SMI, USA)^41^, rinsed and rehydrated in PBS (schematically shown in Fig. 2c). For affinity measurement Fn fibers were deposited onto a pre-stretched sheet and subsequently relaxed to about a strain of 7% as defined earlier^30^. For measurements of the strain-sensitive binding behavior, fibers were either relaxed or stretched (about 380% strain). After a blocking step with BSA (Sigma-Aldrich, Buchs, Switzerland) to avoid unspecific attachment of binding ligands to fibers or to the silicone sheet, the fibers were incubated with varying concentrations of FnBPA5-Alexa-488 or scrambled FnBPA5-Alexa 488 for 1 h and imaged after a washing step by means of confocal microscopy.

### Cell culturing and immunofluorescence stainings

Normal human dermal fibroblasts (PromoCell, Vitaris AG, Switzerland) were cultured in alpha minimum essential medium (α-MEM) with 10% fetal bovine serum from BioWest (Nuaillé, France), and split before reaching confluence. Cells were seeded onto Fn-coated 8-well chambered cover glasses (Nunc^TM^ Lab-Tek^TM^ Chambered Coverglass, Thermo Fisher Scientific, Reinach, Switzerland) at a density of 50 × 10^3^ cells per cm^2^ and allowed to attach to the surface before medium exchange to medium containing 50 μg ml^−1^ Fn. Dependent on the experiment, a fraction of only 10% of exogenous Fn was double-labeled for FRET analysis to prevent intermolecular FRET. Cells in Figs. 2h and 3 were cultured for 48 h. A total of 5 μg ml^−1^ FnBPA5-Alexa488 respectively scrambled FnBPA5-Alexa488 peptide was incubated for 1 h prior to fixation in all cell culture samples. After fixation with a 4% paraformaldehyde solution in PBS, the samples shown in Fig. 3 were stained using DAPI (Invitrogen^TM^, Thermo Fisher Scientific, Switzerland) and were further analyzed by means of confocal microscopy, whereas samples shown in Fig. 2h were blocked by 4% donkey serum (Abcam, Switzerland) for 1 h at room temperature and fibronectin was stained using a rabbit polyclonal anti-fibronectin antibody (1:50 diluted, ab23750, Abcam, Switzerland). Samples shown in Fig. 2h were then incubated for 1 h with a donkey anti-rabbit Alexa-546 secondary antibody (Invitrogen^TM^, Thermo Fisher Scientific, Switzerland).

### FRET analysis

Fn was exogenously added to the cell culture medium at a concentration of 50 μg ml^−1^ with a 10% fraction of double-labeled Fn. Fn is then incorporated by the cells into their matrix^33^. FnBPA5-Cy5 was added 1 h prior to fixation. For FRET analysis, the acceptor fluorescence intensity was divided by donor intensity, pixel-by-pixel to get a FRET ratio with single-pixel resolution. More stretched Fn conformations exhibited lower FRET ratios. FnBPA5 binding was quantified in a similar way, by normalizing the FnBPA5-Cy5 signal with directly excited acceptor emission of Fn. Applied correction factors were assessed in control measurements, presented in Supplementary Fig. 5.

### Confocal microscopy

Single Fn fiber samples were imaged with an Olympus FV 1000 confocal microscope (Olympus AG, Switzerland) using a 40× water immersion objective with a numerical aperture of 0.9. FnBPA5-Alexa-488 and Fn-Cy5 channels were imaged with a 512 × 512 pixel resolution and photomultiplier tube voltage and laser powers were kept constant within an experiment. Fibroblast ECM samples (Fig. 2e) and FRET samples (Fig. 3) were acquired with the same microscope using an oil immersion 1.45 NA 60× objective with a pixel resolution of 1024 × 1024. Images were acquired with the pinhole diameter fixed at 200 μm and pixel dwell time at 8 μs per pixel. Briefly, images were taken from fixed matrices using a 50/50 beam splitter. Samples were excited with a 488 nm laser and acceptor (Alexa-546) and donor (Alexa-488) signal was detected in two PMTs using a 12 nm detection bandwidth for donor (514–526 nm) and acceptor (566–578 nm)^25^. PMT voltage for FRET imaging was kept constant at 550 V for donor and acceptor PMT. Laser transmissivity was adjusted in order to achieve high detection sensitivity while minimizing photobleaching of samples.

### Image analysis

Images were analyzed using Fiji-ImageJ and Matlab (MathWorks, Switzerland). For the Fn fiber affinity study, the pixel-wise ratio of FnBPA5-Alexa- 488 signal intensity divided by Fn-Cy5 intensity was calculated for each fiber, if intensities were above a cut-off threshold and below saturation. A mean of 10 fibers was imaged per experiment and each concentration was done in triplicates. Binding ratio of 10 μM FnBPA5-Alexa-488 concentration was set to 1 and all other points were normalized to this reference, fitted and plot using Origin 7 (OriginLab Corp., Northampton, USA). Statistical analysis was performed using two-tailed type 3 Student *t* test, (Microsoft Excel). The analyses were considered as type 3 (two sample unequal variance) and statistical significance was assumed for *p*-values smaller than 0.05. For ratiometric analysis of fibroblast matrices (Fig. 3), images were smoothed using a 2 × 2 pixel-averaging filter dark current values were subtracted from images and non-realistic extremes and background were excluded from the analysis. Correction factors for donor bleed through and direct activation of the acceptor used for the analysis were experimentally assessed as shown in Supplementary Fig. 5b, c. The ratios of individual channels were then calculated and displayed alongside with a color code for colorimetric illustration of the ratio range of interest. FRET acceptor channel was corrected for donor bleed through which was measured to be ~20% of the acceptor intensity (Supplementary Fig. 5b). Further analysis such as FRET-Fn-FnBPA5/Fn ratio quantifications were carried out in MatLab.

### Fluorescence polarization experiments

The binding affinities of Fn to FnBPA5-Alexa-488 were determined in three independent measurements by anisotropy titrations in a Cary Eclipse Fluorescence Spectrophotometer (Agilent Technologies, Santa Clara, USA) equipped with automated polarizers. FnBPA5 and the scrambled control peptide were synthesized with an N-terminal Alexa 488 dye (piCHEM GmbH, Graz, Austria). The anisotropy of 100 nM Alexa-488 labeled FnBPA5 was measured in PBS at Fn concentrations ranging from 0 to 1.4 μM. Excitation and emission were at *λ*_ex_ 480 nm and *λ*_em_ 520 nm, respectively, with both slit widths set to 10 nm, 20 °C, 5 s signal acquisition and *g* = 1.4. The *K*_d_ values were determined by fitting the data to a one-site-binding model using Origin 7 (OriginLab Corporation, Northampton, USA).

### Preparation and staining of histological tissue sections

Five weeks-old female CD1 nude mice were purchased from Charles River (Germany). After 5 days of acclimatization, the PC-3 tumor cells (human prostate cancer cell line, ACC-465, DSMZ, Braunschweig, Germany) were subcutaneously inoculated in both shoulders of the mice (5 Mio. cells in 100 μL PBS per side). After 4 weeks inoculation, the mice were sacrificed and frozen tumor tissue was cut into 3 μm sections (Microm Cryo-Star HM 560 M, Walldorf, Germany). Non-fixed tissue sections were thawed, rinsed with PBS to wash away all soluble components including plasma Fn, blocked for 30 min with 4% BSA in PBS and incubated for 60 min with 5 μg ml^−1^ FnBPA5-Alexa488 or FnBPA5-Cy5. After a washing step the tissues were fixed in 4% paraformaldehyde in PBS for 10 min. Samples were blocked in PBS with 5% goat serum for 60 min and incubated with polyclonal rabbit anti-fibronectin antibody (ab23750, Abcam, 1:50 dilution) or rat anti CD-31 antibody (ab7388, Abcam, 1:50 dilution) or rabbit anti-alpha SMA antibody (ab5694, Abcam, 1:100 dilution) overnight at 4°C. Primary antibody solution was removed and samples were washed before incubation with secondary goat anti-rabbit Alexa-633 (A-21070, Invitrogen, 1:100 dilution) or goat anti rat Alexa 546 (A-11081, Invitrogen, 1:100 dilution) or goat anti-rabbit Alexa-488 (A-11034, Invitrogen, 1:100 dilution) antibody solution for 45 min. After another washing step, samples were mounted using Prolong Antifade Gold with or without DAPI (Invitrogen). The overview mosaic image in Fig. 6a was acquired using a Nikon TE2000-E epifluorescence microscope equipped with a 10× air objective. Whole tissue section was visualized by stitching together individual fields of view using the grid/collection stitching plugin in Fiji^70^.

Images presented in Fig. 6b were acquired using a Leica SP8 MP microscope. Fn, CD-31 and FnBPA5 were acquired via direct excitation of fluorophores with one photon, whereas SHG was generated using a 900 nm laser and detecting scattered light in a bandwidth around half the incident wavelength (445–460 nm) with an opened pinhole. Images presented in Fig. 4c were acquired using a Leica SP-4 confocal microscope with a 63× oil immersion objective.

### Spatial proximity analysis

Evaluation of spatial proximity of two channels (Fig. 4b, c) was carried out using a custom made Matlab script (MathWorks, Switzerland). High-resolution images as shown in Fig. 4b, c were used, images were smoothed using a 2 × 2 pixel-averaging filter, saturated pixels and background below a specific threshold were furthermore excluded from the analysis. Lower threshold for each individual channel was defined to exclude unspecific signal from analysis. For each pixel in the first channel with an intensity above the lower threshold, a 5 × 5 surrounding evaluation matrix is created looking for proximal pixels in the second channel above the threshold value. In case of one or more pixels in the second channel being above the threshold, the evaluated pixel from the first channel counts as a pixel proximal to the second channel. The values presented in Fig. 4b, c represent the percentage of all pixels of a given channel in proximity to pixels above a threshold of a second channel.

### Radiolabeling of FnBPA5-NODAGA and scrambled derivative

The fibronectin-binding peptide (FnBPA5) and its scrambled control peptide (scrambled FnBPA5) were purchased from Peptide Specialty Laboratories GmbH (Heidelberg, Germany) conjugated with a maleimide NODAGA. The compounds were dissolved in TraceSELECT^®^ Water (Sigma-Aldrich, Buchs, Switzerland) to a final concentration of 0.25 mM. 14 nmol of each peptide were radiolabeled in 0.5 M ammonium acetate pH 5.5 by adding 80 MBq ^111^InCl_3_ (Mallinckrodt, Wollerau, Switzerland) followed by a 30 min incubation step at 50 °C. Quality control was performed by reversed-phase HPLC (Varian Prostar HPLC System, SpectraLab Scientific Inc., Canada); column Dr.Maisch Reprospher 300 C18-TN, 4.6 cm × 150 mm; 5 μm The column was eluted with acetonitrile containing 0.1% TFA and Ultrapure water containing 0.1% TFA and a linear gradient starting with 15% acetonitrile up to 95% over 15 min with a flow rate of 1 mL per minute.

### In vitro plasma stability

To assess in vitro plasma stability 12 MBq ^111^In-FnBPA5 resp. ^111^In-scrambled FnBPA5 were incubated with filtered fresh human blood plasma at 37° C. At different time points (0, 0.25, 0.5, 1, 2, 48, and 72 h) plasma samples were taken out and precipitated by the addition of a solution containing 50% ethanol, 50% acetonitrile, and 0.1% TFA. After that, the samples were filtrated using a Thomson Single StEP Filter vial 0.45 μm PVDF (Thomson Instrument Company, Oceanside, USA) and the supernatant was analyzed by reversed-phase HPLC (Varian Prostar HPLC System, SpectraLab Scientific Inc., Canada) using a D-Bio Discovery Wide Pore C18 column (25 cm × 4.6 mm; 5 μm). The column was eluted with acetonitrile containing 0.1% TFA and Ultrapure water containing 0.1% TFA and a linear gradient starting with 5% acetonitrile up to 95% over 30 min with a flow rate of 1 mL per min.

### Tumor model

All animal experiments were approved by the Cantonal Veterinarian Department of the Canton Aargau (permission number 75500 and 75531) and conducted in accordance with the Swiss law for animal protection. PC-3 cells (human prostate carcinoma cell line, ACC-465, DSMZ, Braunschweig, Germany) were cultured in RPMI 1640 medium (Roswell Park Memorial Institute 1640 medium, Amimed, Bioconcept, Switzerland). Cells were cultured as monolayers at 37 °C in a humidified atmosphere containing 5% CO_2_. The 4–5 weeks-old female CD1 nude mice were purchased from Charles River (Sulzfled, Germany). After 5–7 days acclimatization period, the tumor cells were subcutaneously inoculated in both shoulders of the mice (3 × 10^6^–1 × 10^7^ cells in 100–150 µL PBS per side). Experiments were performed 3–4 weeks after inoculation.

### SPECT/CT imaging and biodistribution studies

SPECT/CT experiments were performed using a 4-head multiplexing multi-pinhole camera (NanoSPECT/CT^plus^, Bioscan Inc., Washington DC, USA). Thirty-three days from the inoculation of the tumor cells, the mice were intravenously injected with ~15 MBq ^111^In-FnBPA5 resp. ^111^In-scrambled FnBPA5-NODAGA (2.4 nmol, 100 µL PBS) into the tail vein. The specific activity of both peptides was 6.2 MBq per nmol. CT scans were performed with a tube voltage of 45 kV and a tube current of 145 µA. SPECT scans at 96 h post injection were obtained post mortem with an acquisition time of 20–90 s. per view resulting in a total scanning time of 20–45 min per mouse. SPECT/CT images after kidney removal were obtained with an acquisition time of ~20 s (^111^In-FnBPA5) and 200 s (^111^In-scrambled FnBPA5) resulting in a total scanning time of 2.5 h for ^111^In-scrambled FnBPA5. SPECT images were reconstructed using HiSPECT software (Scivis GmbH, Goettingen, Germany). The images were reconstituted and processed with InVivoScope^®^ software (Bioscan Inc., Washington DC, USA).

For the biodistribution experiments, PC-3 tumor grafted mice were injected into the tail vein with 150 kBq ^111^In-FnBPA5 or ^111^In-scrambled FnBPA5 (2.4 nmol, 100 µL PBS). For each peptide, groups of four mice were sacrificed at 1, 4, 24, and 96 h post injection. Blocking experiment (*n* = 4) was performed by injection of an excess of peptide (100 µg, 24 nmol in 100 µL PBS) directly before the administration of the corresponding radiolabeled peptide. The organs were collected, weighed and counted for activity with a *γ* counter (Packard Cobra II Auto, Gamma, PerkinElmer AG, Schwerzenbach, Switzerland) 4 h p.i. The results were expressed as a percentage of injected activity per gram of tissue (% IA per g tissue). The Student's t test analyses were considered as type 3 (two sample unequal variance) and statistical significance was assumed for *p*-values smaller than 0.05.

### Data availability

All relevant data are available from the authors upon request.

## Electronic supplementary material

Supplementary Information

Description of Additional Supplementary Files

Supplementary Movie 1

Supplementary Movie 2

Supplementary Movie 3

Supplementary Movie 4
