# Supplementary material for: Novel peptide probes to assess the tensional state of fibronectin fibers in cancer
Source: Nat Commun. 2017 Nov 27;8:1793. doi: 10.1038/s41467-017-01846-0 (PMC5702617; doi:10.1038/s41467-017-01846-0)
Supplement: Supplementary file 1 — Supplementary Information [file 41467_2017_1846_MOESM1_ESM.pdf]

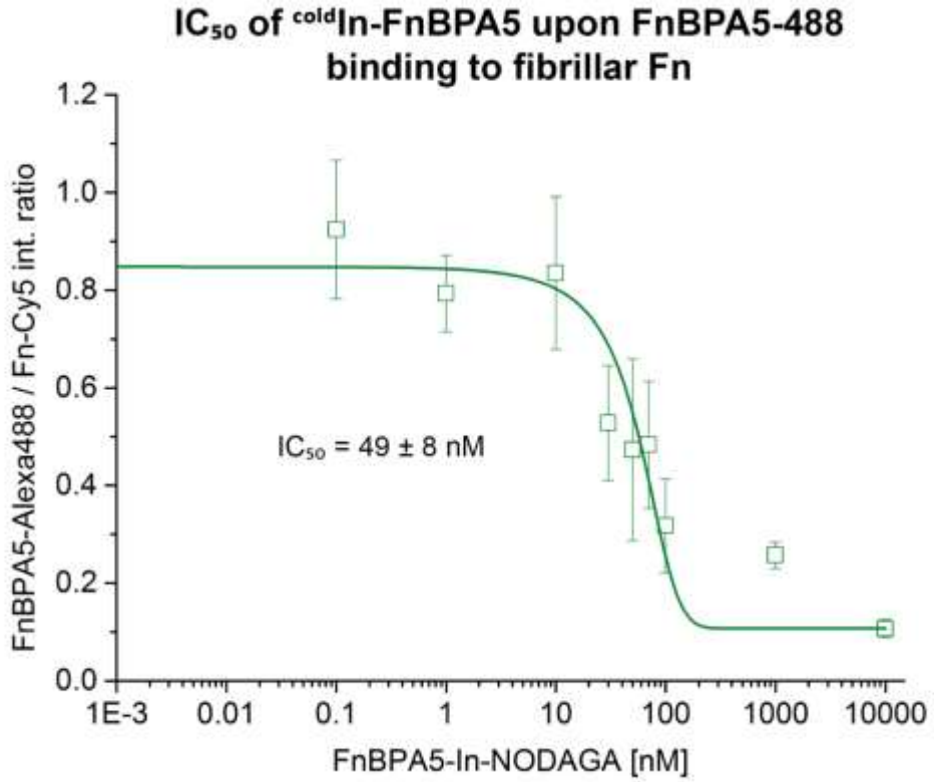

Supplementary Figure 1: IC_{50} measurement of ^{nat}In-FnBPA5 binding to single Fn fibers fabricated as described in the Materials and Methods section. Relaxed Fn fibers were incubated with FnBPA5-Alexa488 peptide, washed and then incubated with different concentrations of In-FnBPA5 to measure the concentration of competitor. At the midpoint between high and low plateau of the curve is the IC_{50} value at 49 ± 8 nM. Points are means from three experiments with error bars representing standard deviations.

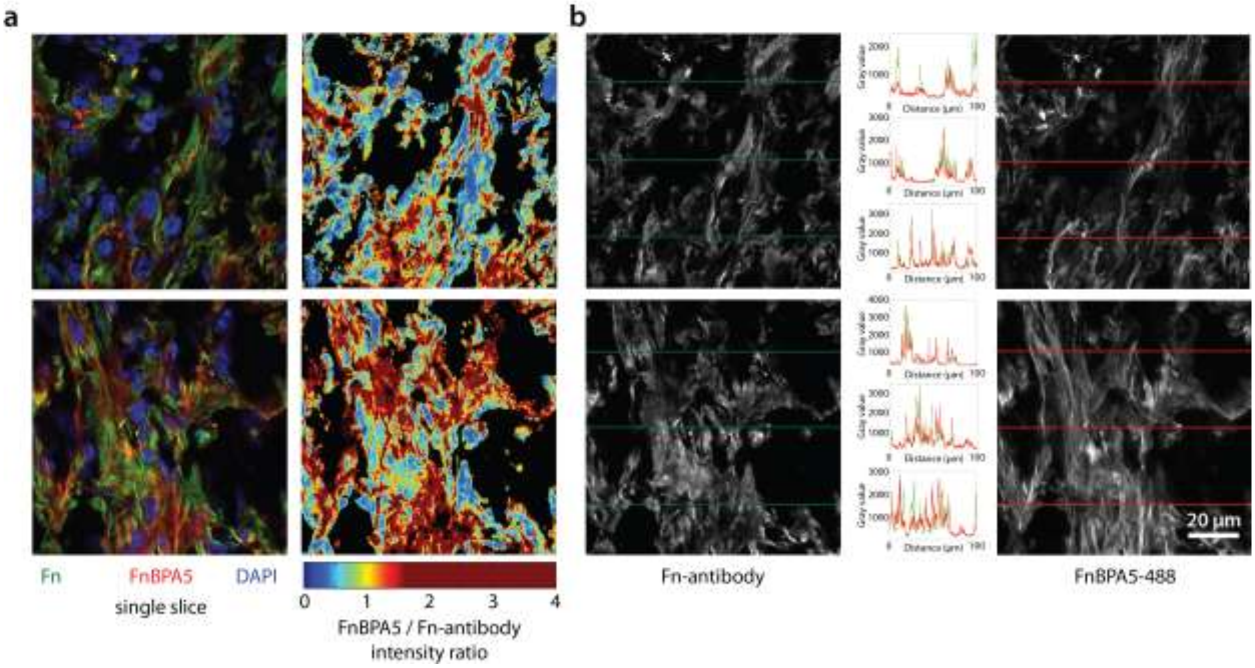

Supplementary Figure 2: High resolution single slice images of FnBPA5 binding to Fn within PC-3 tumor tissue sections. **a** Multi-color image of two representative single-slice high resolution fields of view of PC-3 tumor tissue sections alongside with ratiometric pixel-by-pixel representation of intensity ratios of FnBPA5 / Fn-antibody as quantified from the greyscale images taken at the respective channels from the same fields of view. **b** Corresponding greyscale images shown together with a greyscale intensity line profile analysis for the Fn (green) and FnBPA5 (red) channels along the three lines per image as indicated. .

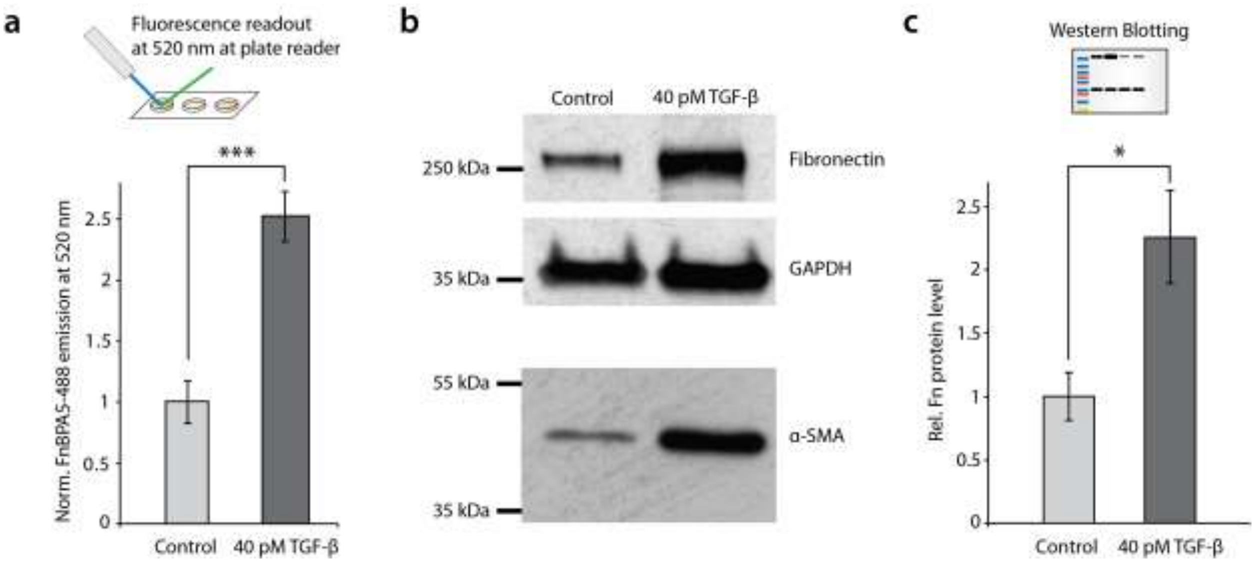

Supplementary Figure 3: Stimulation of human dermal fibroblats with TGF-β in 2D cell culture, measurement of FnBPA5 binding to the native Fn fibrils within the ECM via fluorescence readout using a plate reader and subsequent lysis of cells for quantification of total Fn protein level using Western Blotting. **a** TGF-β treated and control samples were incubated with FnBPA5-488 for 1 hour at 37°C, washed and fluorescence of bound FnBPA5 was measured using a plate reader. TGF-β treated samples exhibited a significant 2.5-fold increase in fluorescence signal from FnBPA5-488 (***$p<0.001$, student's t-test, n=3, bars represent mean values with error bars being standard deviations). After measurement, cells were scraped off the surface and lysed and total protein content was evaluated using a BCA assay. **b** Western blotting was carried out with respective protein lysates, reavealing a significant higher protein level of Fn in the TGF-β treated sample, compared to the control. GAPDH was used as a loading control to normalize for eventual uneven loading of individual wells. Protein levels of alpha smooth muscle actin (αSMA) indicate that TGF-β treatment indeed worked and led to an increase in expression of myofibroblastic marker. **c** Quantification of Western Blots from 3 independent experiments reveal a 2.3-fold, significant increase of Fn level normalized with loading control (*$p<0.05$, student's t-test, n=3, bars represent mean values with error bars being standard deviations), a similar increase in Fn level as the increase in FnBPA5 binding, as observed via plate reader measurements.

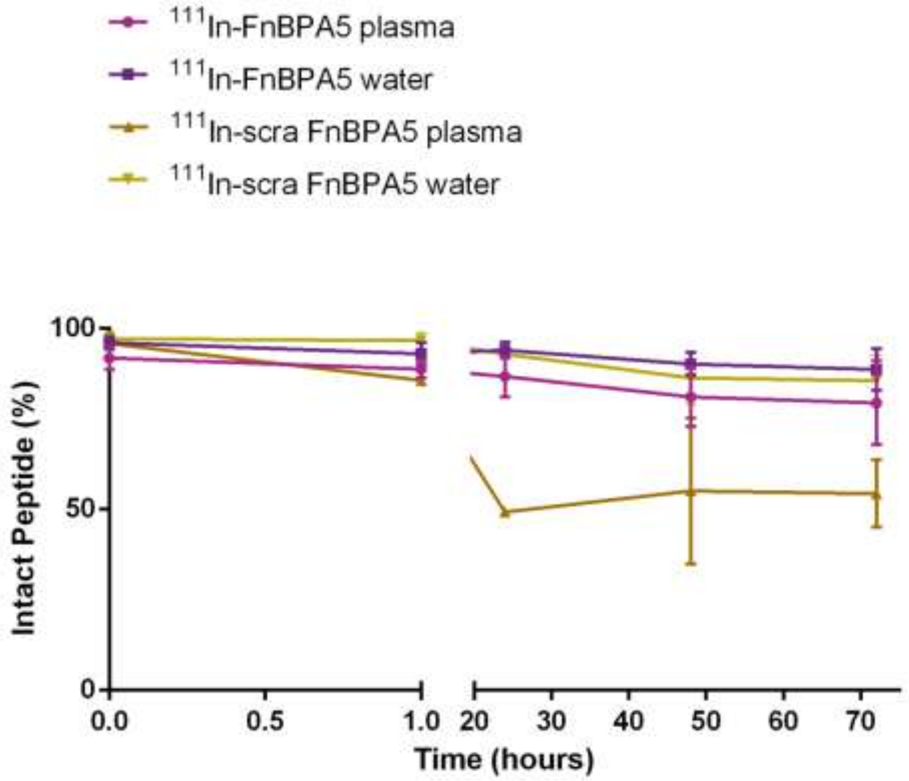

Supplementary Figure 4: Stability of ^{111}In-FnBPA5 and ^{111}In-scrambled FnBPA5 in human blood plasma and water, measured at 37°C at different time points. Both ^{111}In-FnBPA5 and ^{111}In-scraFnBPA5 show high stability at the 1 hour time point. ^{111}In-FnBPA5 shows very high stability also at later time point. Points are means from three experiments with error bars being standard deviations.

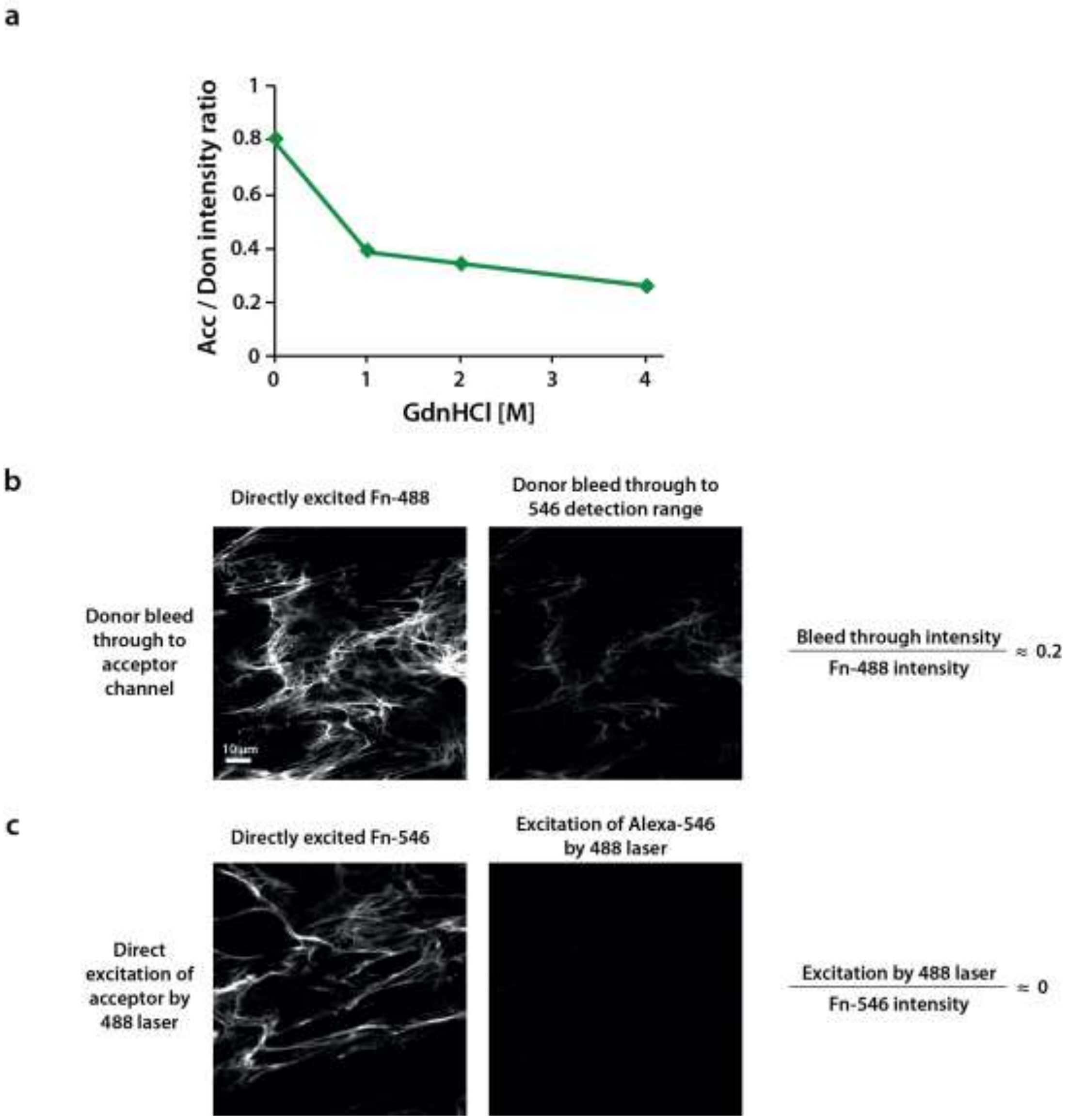

Supplementary Figure 5: Controls for Fn-FRET experiments. **a** Measurements of acceptor/donor intensity ratios of double-labeled Fn in solution under chemical denaturation using increasing concentrations of guanidinium hydrochloride (GdnHCl). Acceptor/donor intensity ratios decrease with increasing concentrations of GdnHCl corresponding to an increased opening of the globular structure of plasma Fn and therefore an increase in average distance between donor-acceptor FRET pair. Acceptor channel has been corrected for donor bleed through before calculation of the FRET ratios. **b** Quantification of donor bleed through to acceptor channel. Donor bleed through to acceptor channel was quantified using a two-day matrix of normal human dermal fibroblasts with exogenous addition of fibronectin to the growth medium (50 μg/ml Fn with 10% single labeled Fn-Alexa 488, randomly labeled at lysines). Quantification of 20 images from 3 different samples showed a 20% bleed through of the donor into the acceptor detection band. The acceptor channel has therefore been corrected in all experiments before calculation of FRET ratio**.** **c** Direct acceptor excitation by 488 laser was quantified similarly, but using single-labeled Fn-Alexa 546 labeled at cysteines. Direct excitation of acceptor by 488 laser was insignificant and neglected in further quantifications.

Supplementary Table 1: Biodistribution data of ^{111}In-FnBPA5 in PC-3 grafted mice

^{111}In-FnBPA5, mean IA% / g ± SD (n = 4)				
Dissection time	1 h p.i.	4 h p.i.	24 h p.i.	96 h p.i.
Blood	1.81 ± 0.62	0.48 ± 0.08	0.15 ± 0.008	0.06 ± 0.027
Heart	1.76 ± 0.51	0.91 ± 0.06	0.31 ± 0.025	0.14 ± 0.017
Lung	2.88 ± 0.64	1.98 ± 0.12	1.05 ± 0.11	0.32 ± 0.038
Spleen	7.01 ± 1.23	3.35 ± 0.33	1.38 ± 0.068	0.73 ± 0.417
Kidneys	140.58 ± 18.10	130.66 ± 10.32	92.36 ± 14.25	39.69 ± 4.79
Pancreas	2.58 ± 1.27	2.31 ± 1.18	0.78 ± 0.47	0.18 ± 0.046
Stomach	3.07 ± 0.39	2.46 ± 0.36	0.51 ± 0.15	0.31 ± 0.052
Intestines	2.27 ± 0.33	1.77 ± 0.12	0.57 ± 0.039	0.13 ± 0.02
Liver	7.52 ± 1.50	3.85 ± 0.16	1.64 ± 0.26	0.89 ± 0.12
Muscle	0.57 ± 0.07	0.50 ± 0.17	0.24 ± 0.034	0.095 ± 0.02
Bone	1.85 ± 0.21	1.32 ± 0.26	0.86 ± 0.117	0.50 ± 0.09
Tumor	4.74 ± 0.77	4.51 ± 0.15	3.59 ± 0.53	1.87 ± 0.773
Tumor-to-blood	3.05 ± 1.65	9.68 ± 1.834	23.93 ± 2.52	34.03 ± 18.36
Tumor-to-liver	0.66 ± 0.22	1.18 ± 0.08	2.21 ± 0.25	2.11 ± 0.82
Tumor-to-kidney	0.03 ± 0.00	0.03 ± 0.00	0.04 ± 0.00	0.05 ± 0.02

Supplementary Table 2: Biodistribution data of the control peptide ^{111}In-scrambled FnBPA5 in PC-3 grafted mice.

^{111}In-scrambled FnBPA5, mean IA% / g ± SD (n = 4)				
Dissection time	1 h p.i.	4 h p.i.	24 h p.i.	96 h p.i.
Blood	0.26 ± 0.064	0.07 ± 0.008	0.04 ± 0.005	0.02 ± 0.005
Heart	0.12 ± 0.03	0.06 ± 0.006	0.04 ± 0.005	0.04 ± 0.005
Lung	0.19 ± 0.03	0.08 ± 0.005	0.05 ± 0.006	0.04 ± 0.014
Spleen	0.21 ± 0.039	0.13 ± 0.036	0.14 ± 0.048	0.10 ± 0.029
Kidneys	163.70 ± 18.90	151.43 ± 9.056	104.62 ± 9.412	23.63 ± 9.883
Pancreas	0.78 ± 0.295	0.64 ± 0.549	0.21 ± 0.162	0.06 ± 0.016
Stomach	0.12 ± 0.013	0.06 ± 0.006	0.04 ± 0.017	0.04 ± 0.029
Intestines	0.09 ± 0.01	0.04 ± 0.012	0.03 ± 0.00	0.02 ± 0.010
Liver	0.10 ± 0.02	0.08 ± 0.026	0.07 ± 0.010	0.05 ± 0.005
Muscle	0.09 ± 0.025	0.04 ± 0.019	0.08 ± 0.026	0.04 ± 0.013
Bone	0.15 ± 0.035	0.10 ± 0.01	0.09 ± 0.021	0.13 ± 0.036
Tumor	0.27 ± 0.08	0.14 ± 0.02	0.09 ± 0.01	0.16 ± 0.08

Supplementary Table 3: Blocking experiment: unlabeled FnBPA5 (24 nmol) was intravenously injected directly before ^{111}In-FnBPA5. The analysis was performed 4 hours p.i. * Significance $p < 0.05$.

^{111}In-labelled FnBPA5, mean IA% / g ± SD (n = 4), blocking experiment		
Dissection time	4 h p.i.	4h p.i. blocking
Blood	0.48 ± 0.08	0.29 ± 0.074*
Heart	0.91 ± 0.06	0.37 ± 0.080*
Lung	1.98 ± 0.12	0.95 ± 0.317*
Spleen	3.35 ± 0.33	1.33 ± 0.402*
Kidneys	130.66 ± 10.32	175.80 ± 32.48
Pancreas	2.31 ± 1.18	0.96 ± 0.384
Stomach	2.46 ± 0.36	1.13 ± 0.14*
Intestines	1.77 ± 0.12	0.53 ± 0.137*
Liver	3.85 ± 0.16	1.61 ± 0.56*
Muscle	0.50 ± 0.17	0.20 ± 0.12*
Bone	1.32 ± 0.26	0.73 ± 0.31*
Tumor	4.51 ± 0.15	2.91 ± 0.70*

Supplementary Table 4: Blocking experiment: unlabeled scrambled FnBPA5 (24 nmol) was intravenously injected directly before ^{111}In-scrambled FnBPA5. The analysis was performed 4 hours p.i., no significant differences were observed

^{111}In-labelled scrambled FnBPA5, mean IA% / g ± SD (n = 4), blocking experiment		
Dissection time	4 h p.i.	4h p.i. blocking
Blood	0.07 ± 0.01	0.07 ± 0.01
Heart	0.05 ± 0.00	0.05 ± 0.01
Lung	0.08 ± 0.01	0.10 ±0.03
Spleen	0.13 ± 0.03	0.18 ± 0.03
Kidneys	151.43 ± 9.06	128.81 ± 17.44
Pancreas	0.64 ± 0.55	1.41 ±1.25
Stomach	0.05 ± 0.01	0.08 ± 0.04
Intestines	0.04 ± 0.01	0.07 ± 0.05
Liver	0.08 ± 0.03	0.08 ± 0.01
Muscle	0.04 ± 0.02	0.04 ± 0.00
Bone	0.09 ± 0.01	0.09 ± 0.01
Tumor	0.14 ± 0.02	0.14 ± 0.04

Supplementary Methods

Fitting of binding curves in Fig. 2d and 2e and Supplementary Figure 1

Fitting assumption was a noncooperative binding of our ligand. It was furthermore assumed that free ligand concentration is the same as the initial ligand concentration ($[L] = [L]_0$).

$$\theta = \frac{[L]}{K_d + [L]}$$

θ … Ratio of occupied binding sites divided by total binding sites
[L]… Free (unbound) ligand concentration
K_d … Dissociation constant

Materials and Methods describing the experiments shown in Supplementary Figure 3

Human dermal fibroblasts were cultured as described in the Method section in the main text and seeded into 12-well plates (TPP, Switzerland) with a density of 50000 cells/well. Cells were allowed to attach for 3 hours before medium exchange to medium with or without 40 pM TGF-β (Peprotech, Switzerland). Medium was changed every second day and cells were stimulated with TGF-β for 5 days. At day 5 samples were incubated with 5 ug/ml FnBPA5-Alexa488 in serum free medium for 1 hour. After incubation cells were washed in PBS and FnBPA5-Alexa488 fluorescence was immediately afterwards measured using a plate reader (Tecan Infinite M200). One sample always acted as negative control and value was subtracted from samples to be analyzed. After plate reader measurement, cells were scraped off and lyzed using a lysis buffer (20mM TRIS (pH 7.5), 150mM NaCl, 1mM EGTA, 1 mM EDTA, 1% Triton-X-100 in PBS) with protease inhibitor cocktail (Roche, Switzerland). Total protein concentration of individual samples were measured by means of BCA protein assay (Thermo Scientific, Switzerland) and plate reader signal was normalized with overall protein concentration. Loading buffer (5x protein loading buffer with 100 mM dithiothreitol) was added to the lysate and protein concentration was adjusted for even loading and heated to 100°C for 5 minutes. Protein samples were then run on a 4-20% gradient polyacrylamide gel (Bio-Rad, Switzerland). Standard running buffer was used (25 mM Tris, 192 mM Glycine, 0.1% SDS). After protein separation by means of SDS-PAGE, the proteins were transferred onto a nitrocellulose membrane (Amersham Biosciences) using standard transfer buffer (25 mM Tris, 192 mM glycine, 20% methanol) and after tranfer blocked in Tris buffered saline (25 mM Tris, 150 mM NaCl) with 4% mild powder. Primary antibodies mouse anti fibronectin antibody clone 10 (610077, BD biosciences, 1:1000 dilution) and rabbit anti GAPDH (G9545, Sigma, 1:1000 dilution) were incubated overnight at 4° C. Secondary donkey anti mouse or donkey anti rabbit antibodies conjugated with HRP (Jackson) were incubated for 1 hour and washed before applying Pierce ECL Western Blotting Substrate (Thermo Scientific) and imaging using Hyperfilm ECL (Amersham Biosciences). Densitometric analysis was carried out using Fiji/ImageJ and subsequently Fn bands were normalized with loading control (GAPDH). After initial membrane staining, membranes were stripped using Restore Western Blot Stripping Buffer (Thermo Scientific) for 10 minutes and stained for mouse anti alpha smooth muscle actin antibody clone 1A4 (A5228, Sigma, 1:1000 dilution) and developed as described above. Statistical analysis was performed using two-tailed type 3 student t-test, (Microsoft Excel). The analyses were considered as type 3 (two sample unequal variance).
