# Supplementary material for: Novel peptide probes to assess the tensional state of fibronectin fibers in cancer
Source: Nat Commun. 2017 Nov 27;8:1793. doi: 10.1038/s41467-017-01846-0 (PMC5702617; doi:10.1038/s41467-017-01846-0)
Supplement: Supplementary file 2 — Description of Additional Supplementary Files [file 41467_2017_1846_MOESM2_ESM.pdf]

Description of Additional Supplementary Files

File Name: Supplementary Movie 1
Description: SPECT/CT rotation video of mouse bearing PC-3 xenografts 96 hours post injection of ^{111}In-FnBPA5, as presented in figure 5A.

File Name: Supplementary Movie 2
Description: SPECT/CT rotation video of mouse bearing PC-3 xenografts 96 hours post injection of ^{111}In-scraFnBPA5, as presented in figure 5B.

File Name: Supplementary Movie 3
Description: SPECT/CT rotation video of mouse bearing PC-3 xenografts with removed kidneys 96 hours post injection of ^{111}In-FnBPA5, as presented in figure 5C.

File Name: Supplementary Movie 4
Description: : SPECT/CT rotation video of mouse bearing PC-3 xenografts with removed kidneys 96 hours post injection of ^{111}In-scraFnBPA5, as presented in figure 5D.
