# Supplementary figures and images for: Novel peptide probes to assess the tensional state of fibronectin fibers in cancer
Source: Nat Commun. 2017 Nov 27;8:1793. doi: 10.1038/s41467-017-01846-0 (PMC5702617; doi:10.1038/s41467-017-01846-0)

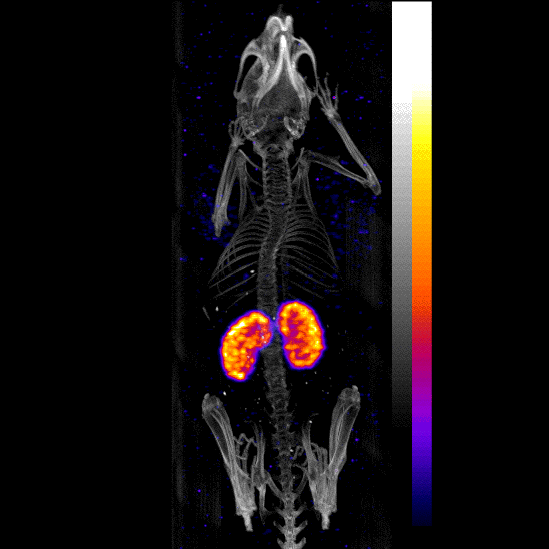

Supplement: Supplementary file 3 — Supplementary Movie 1 [file 41467_2017_1846_MOESM3_ESM.gif]

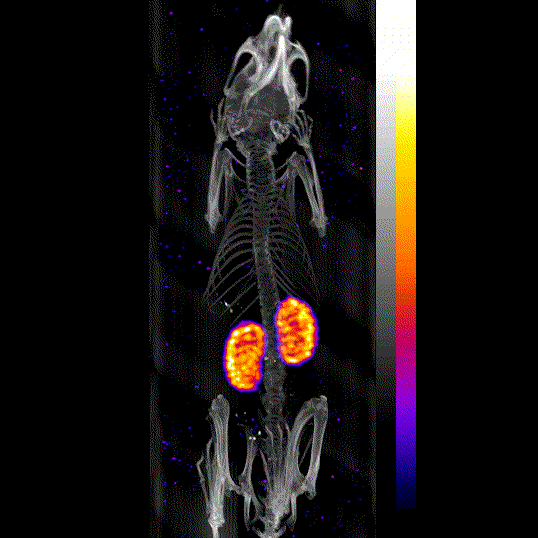

Supplement: Supplementary file 4 — Supplementary Movie 2 [file 41467_2017_1846_MOESM4_ESM.gif]

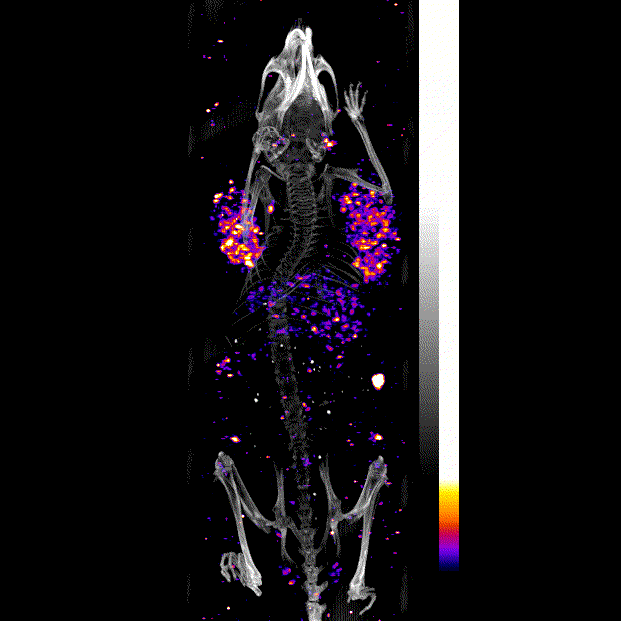

Supplement: Supplementary file 5 — Supplementary Movie 3 [file 41467_2017_1846_MOESM5_ESM.gif]

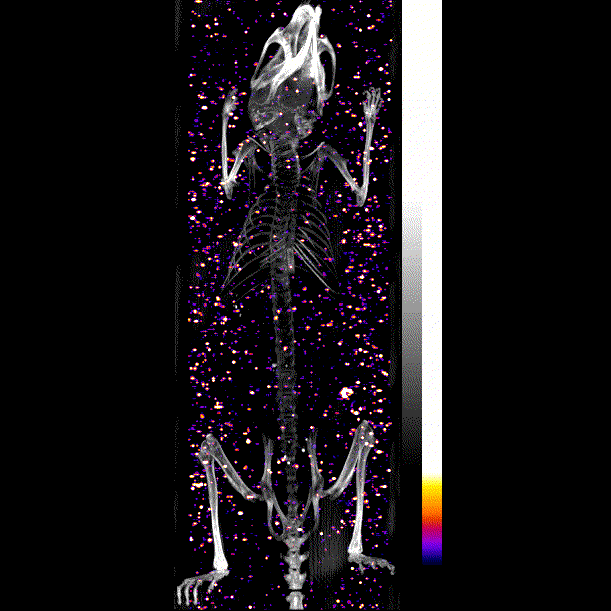

Supplement: Supplementary file 6 — Supplementary Movie 4 [file 41467_2017_1846_MOESM6_ESM.gif]
